# Appropriate waist circumference cut points for identifying insulin resistance in black youth: a cross sectional analysis of the 1986 Jamaica birth cohort

**DOI:** 10.1186/1758-5996-2-68

**Published:** 2010-12-07

**Authors:** Marshall K Tulloch-Reid, Trevor S Ferguson, Novie OM Younger, Jan Van den Broeck, Michael S Boyne, Jennifer M Knight-Madden, Maureen E Samms-Vaughan, Deana E Ashley, Rainford J Wilks

**Affiliations:** 1 Tropical Medicine Research Institute, The University of the West Indies, Mona, Jamaica; 2 Centre for International Health, University of Bergen, Norway; 3 Department of Obstetrics, Gynaecology and Child Health, The University of the West Indies Mona, Jamaica; 4 Institute for Sustainable Development, The University of the West Indies, Jamaica

## Abstract

**Background:**

While the International Diabetes Federation (IDF) has ethnic specific waist circumference (WC) cut-points for the metabolic syndrome for Asian populations it is not known whether the cut-points for black populations should differ from those for European populations. We examined the validity of IDF WC cut points for identifying insulin resistance (IR), the underlying cause of the metabolic syndrome, in predominantly black, young Jamaican adults.

**Methods:**

Participants from a 1986 birth cohort were evaluated between 2005 and 2007 when they were 18-20 years old. Trained observers took anthropometric measurements and collected a fasting blood sample. IR was assessed using the homeostasis model assessment computer programme (HOMA-IR). Sex specific quartiles for IR were generated using HOMA-IR values and participants in the highest quartile were classified as "insulin resistant". Receiver operator characteristic (ROC) curves were used to estimate the best WC to identify insulin resistance. The sensitivity and specificity of these values were compared with the IDF recommended WC cut-points.

**Results:**

Data from 707 participants (315 males; 392females) were analysed. In both sexes those with IR were more obese, had higher mean systolic blood pressure, glucose and triglycerides and lower mean HDL cholesterol. The WC was a good predictor of IR with an ROC area under the curve (95% CI) of 0.71(0.64,0.79) for men and 0.72(0.65,0.79) for women. Using the Youden Index (*J*) the best WC cut point for identifying IR in male participants was 82 cm (sensitivity 45%, specificity 93%, *J *0.38) while the standard cut point of 94 cm had a sensitivity of 14% and specificity of 98% (*J *0.12). In the female participants 82 cm was also a good cut point for identifying IR (sensitivity 52%, specificity 87%, *J *0.39) and was similar to the standard IDF 80 cm cut point (sensitivity 53%, specificity 82%, *J *0.35).

**Conclusions:**

The WC that identified IR in young black men is lower than the IDF recommended WC cut point. Sex differences in WC cut points for identifying IR were less marked in this population than in other ethnic groups.

## Background

Insulin resistance is thought to be the underlying mechanism through which persons develop type 2 diabetes, dyslipidemia and coronary heart disease [1] and when present clusters with other cardiovascular risk factors [2]. Direct measurement of insulin resistance is difficult and therefore in order to help clinicians identify persons who might be insulin resistant a clinically defined entity - the metabolic syndrome - was first proposed by the World Health Organization (WHO) [3]. Subsequently the National Cholesterol Education Program Adult Treatment Panel III (NCEP-ATPIII) and the European Group for the Study of Insulin Resistance (EGIR) formulated their definitions of the syndrome [4,5]. In 2005 the International Diabetes Federation (IDF) proposed a definition of the Metabolic Syndrome that took ethnic differences in waist circumference cut points and additional epidemiologic data into consideration[6]. Despite their differences all recent definitions of the metabolic syndrome have included the same components - glucose intolerance, dyslipidemia (high triglycerides, low HDL-cholesterol), hypertension/elevated blood pressure and central obesity measured using waist circumference.

Central obesity is considered by some to be the factor that drives insulin resistance and the eventual development of metabolic syndrome [7]. In the last few years there has been a worldwide increase in the prevalence of obesity, which includes youth [8]. It is therefore important to develop criteria for central obesity that can identify insulin resistant youth so that preventive measures can be taken before they develop complications of this condition.

The IDF has proposed a definition of the metabolic syndrome in youth for this purpose [7]. It has recommend the use of the Europoid adult criteria in those 16 years and older, regardless of ethnicity, until additional evidence is available to suggest otherwise. These criteria (a waist circumference equal to or greater than 94 cm in men and 80 cm in women) are based on data from a middle aged European population [6]. The appropriateness of this recommendation in middle-aged Black populations is questionable [9-12]. We are not aware of any studies which have examined this issue in young black adults.

The aim of this study was to identify optimum waist circumference cut-points to identify IR among black young adults and to assess the validity of current IDF waist circumference cut points for identifying IR and components of the metabolic syndrome in young black youth.

## Methods

The 1986 Birth Cohort Study consists of a subset of participants from the Jamaican Perinatal Morbidity and Mortality Survey which studied all births in Jamaica during the months of September and October 1986. The details of this study have been described previously [13]. A subset of the cohort was evaluated between 2005 and 2007 when they were between 18 and 20 years old. Participants residing in three parishes in Jamaica (Kingston and St. Andrew or St. Catherine) were identified from contact information at a follow up visit at age 15-16years, newspaper advertisements and visits to their last known address. Approval for the study was obtained from The University of the West Indies Ethics Committee. All subjects were evaluated at the Tropical Medicine Research Institute after a 10 hour fast. Written informed consent was obtained from each participant at the time of their visit to the Institute.

Trained nurses performed the anthropometric and blood pressure measurements. Weight was measured to the nearest 0.1 kg with an electronic digital scale (Tanita HD-316, Japan). Height was measured to the nearest 0.1 cm using a portable height measurement rod. Waist circumference was measured to the nearest 0.1 cm using a non-stretchable tape at the narrowest circumference between the lowest rib and the anterior superior iliac crest. Sitting blood pressure was measured with a mercury sphygmomanometer to the nearest 2 mm Hg using the first (systolic) and fifth (diastolic) Korotkoff phases using a standardized protocol [14]. Three measurements were taken at 1-minute intervals after the participant had been seated for five minutes and the mean of the last two readings was used for the analysis.

The fasting blood specimens were collected in plain, fluoridated and heparinized tubes by the research nurses and processed and stored as appropriate at the Tropical Medicine Research Institute Laboratory within three hours of collection. Insulin was measured using a chemiluminesent immunometric assay (Immulite, Diagnostic Products Corporation, Los Angeles, CA) from a serum sample. The assay had an analytical sensitivity of 13.9 pmol/L, little cross reaction with pro-insulin and the intra-assay coefficient of variation was < 8.0%. Plasma glucose was measured using the glucose oxidase method (Alcyon). Insulin resistance (HOMA-IR) was assessed using homeostasis model assessment computer programme HOMA2 (from the Oxford University Diabetes Trials Unit Website at http://www.dtu.ox.ac.uk/homa) incorporating the fasting insulin and glucose values. Higher values of HOMA-IR corresponded to more severe insulin resistance [15]. Total cholesterol, HDL-cholesterol and triglycerides were measured directly (Abbot Spectrum) and LDL-cholesterol calculated using the Friedewald equation [16].

### Statistical Analysis

Subjects were defined as having IR if they were in the highest sex specific fasting HOMA quartile - an approach taken by the EGIR for defining hyperinsulinemia [5]. Comparisons of continuous variables between insulin resistant and non-insulin resistant subjects by sex were made using the Student's t test or Wilcoxon Rank Sum Test for variables that were not normally distributed. Sex specific Receiver Operating Characteristic (ROC) curves were used to estimate sensitivity and specificity of different waist circumference values to identify participants with IR. Sensitivity and specificity were computed assuming that each variable was positively related to the presence of insulin resistance. The ROC Curves were then used to determine the cut point with the highest sensitivity and specificity for identifying insulin resistance in each sex. For each waist circumference cut point the Youden Index (*J *) was also calculated to confirm that this was the optimal cut point. The Youden Index is calculated as the (sensitivity + specificity) - 1 and ranges from 0 and 1 [17]. The optimal cut point occurs where the *J *value is closest to 1. The Youden index has been demonstrated to be an optimal means of selecting cut points and in some instances may be preferred to visual identification of the optimal cut point from the ROC curve [18]. The sensitivity and specificity of the IDF waist circumference cut points for identifying insulin resistance were also determined.

The ability of the waist circumference to identify young adults with the other components of the metabolic syndrome - low HDL cholesterol (< 1.0 mmol/L in men and 1.3 mmol/L in women), high triglycerides (> 1.7 mmol/L), high glucose (> 5.6 mmol/L) and elevated blood pressure (systolic BP >130 mmHg and/or diastolic BP >85 mmHg) was also assessed. The sensitivity and specificity of the study derived waist circumference cut points for identifying these components were then compared with the sex specific IDF waist circumference cut points.

Analysis was performed using Stata 8.0 (Stata Corporation, College Station TX).

## Results

A total of 707 participants (315 men 392 women), none of whom had diabetes (fasting plasma glucose > 7.0 mmol/L or taking medications for diabetes), had data available for all the variables of interest and a HOMA-IR measurement. The characteristics of the subjects by sex and their IR status using HOMA IR are presented in Table 1. Male participants classified as insulin resistant had higher systolic blood pressure, waist circumference, fasting glucose, triglycerides and LDL cholesterol and lower HDL cholesterol than men who were not insulin resistant. Results for women were similar but there was no statistically significant difference in LDL cholesterol between women with and without IR. There were no significant differences in diastolic blood pressure in either sex by IR status.

**Table 1:** Characteristics of the study population by sex and insulin resistance category (highest sex-specific HOMA-IR quartile)

Characteristic	Men		Women	
	**Insulin Resistant** **(n = 74)**	**Insulin Sensitive** **(n = 241)**	**Insulin Resistant** **(n = 81)**	**Insulin Sensitive** **(n = 311)**
Age (years)	19.3 ± 0.5	19.3 ± 0.5	19.3 ± 0.5	19.4 ± 0.5
Systolic Blood Pressure (mmHg)^a, d^	115 ± 11	113 ± 10	110 ± 8	107 ± 8
Diastolic Blood Pressure (mmHg)	71 ± 10	69 ± 11	67 ± 9	67 ± 9
Body Mass Index (kg/m^2^)^b, d^	25.5 ± 6.3	21.7 ± 3.1	27.5 ± 7.5	22.1 ± 4.5
Waist Circumference (cm)^b, d^	80 ± 14	73 ± 9	83 ± 16	71 ± 10
Fasting glucose (mmol/L)^b, d^	4.9 ± 0.5	4.7 ± 0.4	4.6 ± 0.4	4.4 ± 0.4
Total Cholesterol (mmol/L)^b^	4.4 ± 1.0	4.0 ± 0.7	4.4 ± 0.9	4.5 ± 0.8
HDL-cholesterol (mmol/L)^a, d^	1.1 ± 0.2	1.2 ± 0.3	1.2 ± 0.3	1.3 ± 0.3
Triglycerides (mmol/L)^b, c^	0.7 ± 0.4	0.6 ± 0.2	0.6 ± 0.3	0.5 ± 0.2
LDL-cholesterol (mmol/L)^b^	3.0 ± 0.9	2.6 ± 0.7	3.0 ± 0.9	2.9 ± 0.7
HOMA-IR†^b, d^	1.2 (1.1, 1.5)	0.6 (0.5, 0.7)	1.8 (1.5, 2.0)	0.8 (0.6, 1.0)

^a ^*P *< 0.05 in men, ^b ^*P *< 0.01 in men ^c ^*P *< 0.05 in women, ^d ^*P *< 0.01 in women † - Median (25^th^-75^th ^centile) - comparisons made using Wilcoxon Test

**Figure 1:**
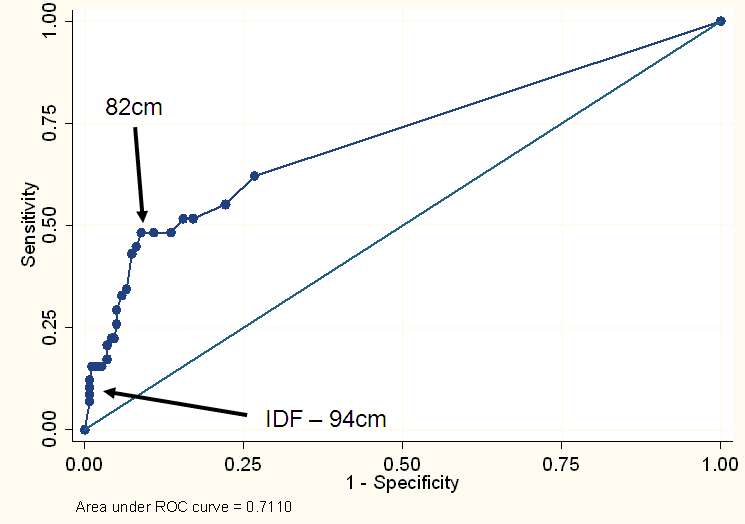
**Receiver Operating Characteristic curve for the ability of the waist circumference to identify men with insulin resistance (top sex-specific quartile based on HOMA-IR)**.

**Figure 2:**
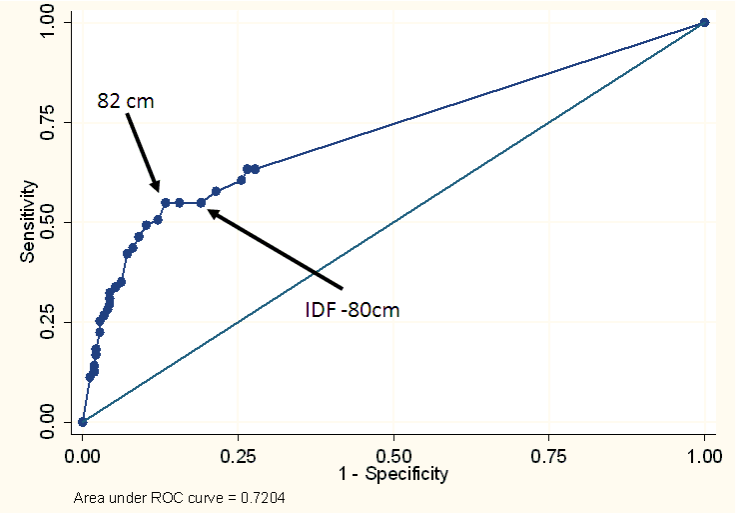
**Receiver Operating Characteristic curve for the ability of the waist circumference to identify women with insulin resistance (top sex-specific quartile based on HOMA-IR)**.

The waist circumference was better than chance in predicting IR in both the male and female participants with a ROC area under the curve (95%CI) of 0.68 (0.61, 0.75) in male participants and 0.71 (0.65, 0.77) in the female participants. (See Figures 1 and 2)

Sex-specific cut points for waist circumference (in 1 cm increments) and the Youden Index for each potential cut point were determined. In both sexes a waist circumference of 82 cm provided the highest sensitivity and specificity for identifying IR (Please see Table 2). The sensitivity for the study derived waist circumference cut point was higher than that of standard IDF criteria in men (45% vs 14%), suggesting that the IDF criterion might fail to identify IR young black men. In women there was little difference in sensitivity between the IDF and standard criteria (53 vs 52% respectively), though in this study the 82 cm cut point had a higher specificity (87% vs 82%) and Youden Index (0.39 vs 0.35) than the 80 cm proposed by IDF. The study derived waist circumference cut points remained unchanged when persons in the highest tertile, quintile or decile were defined as insulin resistant.

**Table 2:** Sensitivity and Specificity of study-derived and IDF recommended waist circumference cut points for identifying insulin resistance in male and female young black adults

Waist Circumference	Sensitivity (%)	Specificity (%)	Youden Index (*J*)
**Men**			
Study derived (82 cm)	45	93	0.38
IDF Standard (94 cm)	14	98	0.12
**Women**			
Study derived (82 cm)	52	87	0.39
IDF Standard (80 cm)	53	82	0.35

Waist circumference was better than chance in identifying men and women with low HDL and men with high triglycerides, but was not better than chance in identifying elevated blood pressure or fasting glucose in any of the young adults. The sensitivity and specificity of the 82 cm and IDF recommended waist circumference cut points for identifying participants with the other metabolic abnormalities is presented in Table 3. In men the IDF recommended waist circumference had a low sensitivity for identifying metabolic abnormalities.

**Table 3:** Sensitivity and Specificity of the study-derived and IDF waist circumference cut points for identifying components of the Metabolic Syndrome by sex.

	Men (n = 315)			Women (n = 392)		
	Proportion affected (%)	IDF 94 cm cut point Sensitivity/Specificity	82 cm cut point Sensitivity/Specificity	Proportion affected (%)	IDF 80 cm cut point Sensitivity/Specificity	82 cm cut point Sensitivity/Specificity
**Elevated blood pressure (> 130/85)**	10	_	_	2	_	_
**Low HDL cholesterol (< 1.3 mmol/L women or <1.1 mmol/L men)**	30	9/98	26/89	62	29/85	24/88
**Elevated fasting glucose** **(> 5.6 mmol/L)**	2	_	_	0.5	_	_
**Elevated fasting triglycerides (> 1.7 mmol/L)**	0.6	50/100	0/100	0.3	_	_

Variables where waist circumference was not better than chance in identifying the presence of a particular metabolic abnormality are left blank

IDF - International Diabetes Federation

## Discussion

In this study of predominantly black young adults the IDF waist circumference failed to identify the majority of male subjects with insulin resistance measured by HOMA-IR. The optimal cut point of 82 cm derived from the study was much lower than that proposed by the IDF in men suggesting the need to lower this value to improve the sensitivity to identify those with metabolic abnormalities. The IDF criteria performed much better in the female participants and were very similar to those derived from this study. Unlike other populations the study derived cut-points for waist circumference to identify insulin resistance were similar in young black men and women.

There are limited data on ideal cut points for the identification of insulin resistant persons of African decent. In a cross sectional study of black populations in Cameroon, Nigeria, Jamaica, St. Lucia and Barbados the waist circumference cut points for identifying persons with hypertension were lower than the IDF criteria and sex differences in these cut points were not as large as for other populations [19]. In a subsequent cohort study of middle aged Jamaicans a waist circumference of 88 cm in men and 84.5 cm in women was found to be the optimal cut points for predicting incident diabetes [9]. A cross sectional analysis of US adults using NHANES data demonstrated racial differences in the waist circumference cut points that correspond to a BMI of 25 kg/m^2^, - the basis of the cut points used to identify those with metabolic abnormalities. In the Black Americans the waist circumferences corresponding to a BMI of 25 kg/m^2 ^were 86.4 cm in men and 83.5 cm in women compared with 91.3 cm and 83.4 cm in White American men and women respectively [11]. While the waist circumference corresponding to a BMI of 25 kg/m^2 ^was about 5-6 cm higher in white men compared to black men, there was no significant racial difference in the waist circumference corresponding to a BMI of 25 kg/m^2 ^in the women. These studies suggest the need for additional data to develop more appropriate cut points for Black populations which may differ by age and sex. Similar to the findings of this study the sex differences in the waist circumference cut points among blacks in all three studies were not pronounced.

The IDF criteria for the diagnosis of the metabolic syndrome in adolescents was an attempt to standardize how the disease was defined in younger populations worldwide. Unlike the criteria for adults no consideration has been given to racial differences in the waist circumference that confer additional risk. Our data suggests the need for lower cut points in young black populations compared to middle-aged European populations in order to identify those with insulin resistance.

The study derived waist circumference cut point in men improved sensitivity for detecting insulin resistant young adults, however the sensitivity still remained low. In both men and women the study derived waist circumference cut point identified only about half of those who were insulin resistant. A lower cut point could be considered but this would reduce the specificity of the measurement and could result in unnecessary expenditure in assessment and treatment of young adults who are not at higher risk of complications. The final decision on the waist circumference cut point used by any health system should ultimately be determined by the available human, infrastructural and economic resources.

The low sensitivity of waist circumference for identifying insulin resistant young black men and women suggests that central obesity may need to be combined with other abnormalities to improve the ability to identify those with this condition. Our findings support the recent recommendation by the IDF that abdominal obesity should no longer be a prerequisite for the diagnosis of metabolic syndrome but one of several abnormalities that constitute the metabolic syndrome [20].

The waist circumference was better than chance in identifying low HDL-cholesterol in both sexes and elevated triglycerides only in men. Waist circumference was not better than chance in identifying the other components of the metabolic syndrome. This may have been a result of the low prevalence of some of these abnormalities. For example elevated fasting glucose and elevated triglycerides were present in less than 2 percent of the population. The study also highlights the differences in the metabolic abnormalities in Caribbean black populations which are typically characterized by a high prevalence of "low" HDL-cholesterol and a low prevalence of elevated triglycerides [21,22].

This study had some limitations. In this analysis we utilized a HOMA-IR score derived from a computer program as the method for the measurement of insulin resistance [15] and not insulin resistance measured by clamp or insulin sensitivity by the minimal model. The model used in the HOMA-IR calculation was derived from non-linear empirical equations producing an algebraic solution and this is more accurate than measurements derived graphically or from simple mathematical approximations.

As there is no standardized definition of insulin resistance we used the distribution of the HOMA-IR scores from the population to define insulin resistance and classified those in the highest HOMA-IR sex specific quartile as insulin resistant. As a consequence the absolute definition of insulin resistance is likely to differ from that used for middle-aged populations and this may influence the definition of waist cut-points. The decision to define insulin resistance this way was based on the approach taken by the EGIR to define hyperinsulinemia as being in the top quartile of fasting insulin values in a non-diabetic population [5]. We tested differing definitions of insulin resistance based on the HOMA-IR. Defining those in the top tertile, quintile or decile as insulin resistant did not affect the findings from the analysis. Additionally the sample consisted of young adults and it may not be appropriate to extrapolate our findings to older individuals.

In this cross sectional analysis of a young adult population the ability of these waist circumference cut points to predict more established outcomes of insulin resistance such as coronary disease could not be assessed. If we are able to re-examine this cohort, data on intermediate outcomes of cardiovascular disease such as carotid intima-media thickness or endothelial reactivity can also be collected. We are not aware of any other studies of black youth from the Caribbean (a region that is currently undergoing the epidemiologic transition) with detailed laboratory measurement of metabolic abnormalities and insulin resistance.

## Conclusions

The IDF waist circumference criteria have poor sensitivity to identify insulin resistance in young black men but perform better among young black women. From our data a waist circumference of 82 cm could potentially be used to identify insulin resistance and some metabolic abnormalities in both young Jamaican men and women.

## Competing interests

The authors declare that they have no competing interests.

## Authors' contributions

All authors have read and approved the final version of this manuscript. MTR - contributed to data collection, data analysis, writing and critical review of the manuscript, TSF - supervised field activities for data collection, contributed to data-analysis strategies and critically reviewed the manuscript, NOMY - contributed to data collection, data analysis and critical review of the manuscript, JVDB - contributed to data collection and quality assurance and control, and critical review of the manuscript. MSB - contributed to data collection and interpretation and critical review of the manuscript, JMKM - contributed to data collection and interpretation and critical review of the manuscript, MESV - contributed to the design of the study and critical review of the manuscript, DEA - contributed to the design of the study and critical review of the manuscript, RJW - conceived and designed the study and directed its implementation including quality assurance and control, contributed to the data analysis strategies and data interpretation, critically reviewed drafts of the manuscripts

## References

[B1] Reaven GM Banting lecture 1988. Role of insulin resistance in human disease Diabetes 1988 37 1595 1607 10.2337/diabetes.37.12.1595 3056758

[B2] Eckel RH Grundy SM Zimmet PZ The metabolic syndrome Lancet 2005 365 1415 1428 10.1016/S0140-6736(05)66378-7 15836891

[B3] Alberti KG Zimmet PZ Definition, diagnosis and classification of diabetes mellitus and its complications. Part 1: diagnosis and classification of diabetes mellitus provisional report of a WHO consultation Diabet Med 1998 15 539 553 10.1002/(SICI)1096-9136(199807)15:7<539::AID-DIA668>3.0.CO;2-S 9686693

[B4] Executive Summary of The Third Report of The National Cholesterol Education Program (NCEP) Expert Panel on Detection, Evaluation, And Treatment of High Blood Cholesterol In Adults (Adult Treatment Panel III) JAMA 2001 285 2486 2497 10.1001/jama.285.19.2486 11368702

[B5] Balkau B Charles MA Comment on the provisional report from the WHO consultation. European Group for the Study of Insulin Resistance (EGIR) Diabet Med 1999 16 442 443 10.1046/j.1464-5491.1999.00059.x 10342346

[B6] Alberti KG Zimmet P Shaw J The metabolic syndrome--a new worldwide definition Lancet 2005 366 1059 1062 10.1016/S0140-6736(05)67402-8 16182882

[B7] Zimmet P Alberti G Kaufman F Tajima N Silink M Arslanian S Wong G Bennett P Shaw J Caprio S The metabolic syndrome in children and adolescents Lancet 2007 369 2059 2061 10.1016/S0140-6736(07)60958-1 17586288

[B8] Low S Chin MC Deurenberg-Yap M Review on epidemic of obesity Ann Acad Med Singapore 2009 38 57 59 19221672

[B9] Sargeant LA Bennett FI Forrester TE Cooper RS Wilks RJ Predicting incident diabetes in Jamaica: the role of anthropometry Obes Res 2002 10 792 798 10.1038/oby.2002.107 12181388

[B10] Sumner AE Sen S Ricks M Frempong BA Sebring NG Kushner H Determining the waist circumference in african americans which best predicts insulin resistance Obesity (Silver Spring) 2008 16 841 846 10.1038/oby.2008.11 18292752

[B11] Zhu S Heymsfield SB Toyoshima H Wang Z Pietrobelli A Heshka S Race-ethnicity-specific waist circumference cutoffs for identifying cardiovascular disease risk factors Am J Clin Nutr 2005 81 409 415 15699228 10.1093/ajcn.81.2.409

[B12] Bouguerra R Alberti H Smida H Salem LB Rayana CB El Atti J Achour A Gaigi S Slama CB Zouari B Alberti KG Waist circumference cut-off points for identification of abdominal obesity among the tunisian adult population Diabetes Obes Me tab 2007 9 859 868 10.1111/j.1463-1326.2006.00667.x 17924868

[B13] Ashley D McCaw-Binns A Foster-Williams K The perinatal morbidity and mortality survey of Jamaica 1986-1987 Paediatr Perinat Epidemiol 1988 2 138 147 10.1111/j.1365-3016.1988.tb00194.x 3237494

[B14] Ataman SL Cooper R Rotimi C McGee D Osotimehin B Kadiri S Kadiri S Kingue S Muna W Fraser H Forrester T Wilks R Standardization of blood pressure measurement in an international comparative study J Clin Epidemiol 1996 49 869 877 10.1016/0895-4356(96)00111-4 8699206

[B15] Levy JC Matthews DR Hermans MP Correct homeostasis model assessment (HOMA) evaluation uses the computer program Diabetes Care 1998 21 2191 2192 10.2337/diacare.21.12.2191 9839117

[B16] Friedewald WT Levy RI Fredrickson DS Estimation of the concentration of low-density lipoprotein cholesterol in plasma, without use of the preparative ultracentrifuge Clin Chem 1972 18 499 502 4337382

[B17] Youden WJ Index for rating diagnostic tests Cancer 1950 3 32 35 10.1002/1097-0142(1950)3:1<32::AID-CNCR2820030106>3.0.CO;2-3 15405679

[B18] Perkins NJ Schisterman EF The inconsistency of "optimal" cutpoints obtained using two criteria based on the receiver operating characteristic curve Am J Epidemiol 2006 163 670 675 10.1093/aje/kwj063 16410346 PMC1444894

[B19] Okosun IS Rotimi CN Forrester TE Fraser H Osotimehin B Muna WF Cooper RS Predictive value of abdominal obesity cut-off points for hypertension in blacks from west African and Caribbean island nations Int J Obes Relat Metab Disord 2000 24 180 186 10.1038/sj.ijo.0801104 10702768

[B20] Alberti KG Eckel RH Grundy SM Zimmet PZ Cleeman JI Donato KA Fruchart JC James WP Loria CM Smith SC Jr Harmonizing the metabolic syndrome: a joint interim statement of the International Diabetes Federation Task Force on Epidemiology and Prevention; National Heart, Lung, and Blood Institute; American Heart Association; World Heart Federation; International Atherosclerosis Society; and International Association for the Study of Obesity Circulation 2009 120 1640 1645 10.1161/CIRCULATIONAHA.109.192644 19805654

[B21] Ferguson TS Tulloch-Reid MK Younger NO Knight-Madden JM Samms-Vaughan M Ashley D Van den Broeck J Wilks RJ Prevalence of the metabolic syndrome and its components in relation to socioeconomic status among Jamaican young adults: a cross-sectional study BMC Public Health 2010 10 307 10.1186/1471-2458-10-307 20525300 PMC2898824

[B22] Ferguson TS Younger N Tulloch-Reid MK Forrester TE Cooper RS Van den Broeck J Wilks RJ Prevalence of the Metabolic Syndrome in Jamaican Adults and its Relationship to Income and Education Levels West Indian Med J 2010 59 265 273 21291104

